# Silent Twiddler syndrome despite dual-anchor fixation in deep brain stimulation: two case reports

**DOI:** 10.3389/fnins.2026.1885621

**Published:** 2026-07-10

**Authors:** Michał Sobstyl, Piotr Glinka, Karol Karamon, Paweł Gogol, Beniamin Oskar Grabarek

**Affiliations:** 1 Department of Neurosurgery, Institute of Psychiatry and Neurology, Warsaw, Poland; 2 Department of Radiology, Institute of Psychiatry and Neurology, Warsaw, Poland; 3 Pain Treatment Clinic, Our Lady of Perpetual Help Hospital, Wołomin, Poland; 4 Collegium Medicum, WSB University, Dabrowa Gornicza, Poland

**Keywords:** deep brain stimulation, extension lead twisting, hardware complication, implantable pulse generator, Twiddler syndrome

## Abstract

**Background:**

Twiddler syndrome (TS) is a rare hardware-related complication in which manipulation or excessive mobility of an implanted pacemaker or implantable pulse generator (IPG) results in device malfunction. Traditionally, TS is diagnosed only after lead coiling, fracture, displacement, or loss of therapeutic efficacy, suggesting that it represents a late-stage manifestation of the condition.

**Cases:**

We report 2 patients with deep brain stimulation (DBS) systems who presented with a subjective sensation of increased IPG mobility during changes in body position. Neither patient demonstrated mechanical failure, lead displacement, or deterioration of DBS clinical benefit, and both IPGs had been implanted with two anchoring sutures fixed to the fascia. Furthermore, neither patient reported intentional manipulation of the device. Because of persistent discomfort and abnormal mobility, revision surgery was performed. Intraoperative findings confirmed early-stage TS in both cases. The IPGs were reimplanted into tailored subpectoral pockets and firmly secured to the fascia. Following revision, both patients experienced complete resolution of symptoms and maintained sustained therapeutic benefit from DBS.

**Conclusion:**

These cases suggest that a subjective perception of increased IPG mobility may represent a distinct pre-Twiddler clinical syndrome that precedes the development of classic TS and overt hardware failure. Early recognition of this underrecognized presentation and timely surgical intervention through pocket revision and device immobilization may prevent progression to lead damage, device malfunction, and serious neurological adverse events, while ensuring uninterrupted neuromodulation therapy.

## 1 Introduction

Twiddler syndrome (TS) is a rare hardware-related complication of implantable pulse generator (IPG) systems, characterized by spontaneous rotation or intentional manipulation of the device within the subclavicular or abdominal pocket, leading to twisting, displacement, or damage of extension leads (Bayliss et al., 1968). TS has been reported in patients treated with deep brain stimulation (DBS) for movement disorders, epilepsy, and neuropsychiatric disorders (Gelabert-Gonzalez et al., 2010). In its classical form, TS usually presents with hardware malfunction manifested by loss of stimulation efficacy, recurrence of neurological symptoms, abnormal impedance measurements, or lead fracture (Bayliss et al., 1968; Gelabert-Gonzalez et al., 2010; Israel and Spivak, 2008; Machado et al., 2005).

TS is most commonly associated with intentional or unconscious manipulation of the IPG, particularly in patients with psychiatric comorbidities or excessive mobility of the implanted device (Franzini et al., 2018). Additional predisposing factors include advanced age, obesity, lax subcutaneous tissue, and creation of an excessively large IPG pocket (Permana et al., 2024). Hardware-related factors may also contribute to TS development, as inadequate fixation of the IPG may facilitate rotational movement within the pocket. The introduction of modern IPGs with dual anchoring holes and improved fixation techniques has been associated with a reduced incidence of TS in recent years (Burdick et al., 2010).

Despite these preventive strategies, we identified TS in 2 patients implanted with modern DBS systems using dual-anchor fixation of the IPG to the fascia. In both cases, stimulation efficacy and impedance measurements remained normal, and neither patient reported intentional manipulation of the device. The initial manifestations were limited to progressive IPG mobility and discomfort at the implantation site. These cases suggest that increased IPG mobility may represent an early - silent clinical stage of TS preceding overt hardware failure.

To our knowledge, these are among the first reported cases clinically silent TS occurring despite dual-anchor IPG fixation and preserved DBS efficacy. Unlike classical TS, both patients initially presented only with abnormal IPG mobility and local discomfort without stimulation failure or abnormal impedance values. These observations expand the clinical spectrum of TS and suggest that subtle mechanical symptoms may precede overt hardware dysfunction.

## 2 Case presentations

A chronological summary of the clinical course, diagnostic assessment, and therapeutic interventions in both patients is presented in Table 1.

**Table 1:** Clinical timeline of the reported cases.

Timepoint	Case 1	Case 2
Initial diagnosis	Medically refractory essential tremor	Medically refractory essential tremor
DBS implantation	Unilateral DBS implantation	Bilateral DBS implantation
DBS target	Ventral intermediate nucleus of the thalamus	Posterior subthalamic area
Initial postoperative outcome	Significant tremor reduction	Significant tremor reduction
First abnormal symptoms	Progressive IPG mobility and discomfort after 2 years	Abnormal positional IPG mobility after 2 months
Physical findings	IPG rotation up to 90° within the pocket	Excessive IPG mobility with tenderness along extension cables
DBS efficacy	Preserved	Preserved
Impedance measurements	Normal	Normal
Preoperative diagnosis	Mechanical instability suspected	Early hardware instability suspected
Revision surgery findings	Severe extension lead twisting and disrupted fixation suture	Severe extension lead coiling and twisting
Surgical intervention	Lead untwisting and subpectoral IPG replacement	Extension lead replacement and subpectoral rechargeable IPG implantation
Follow-up outcome	Resolution of pain and abnormal mobility	Resolution of pain and abnormal mobility
Follow-up duration	8 months	8 months

### 2.1 Case 1

A 72-year-old woman with medically refractory essential tremor (ET) underwent left-sided deep brain stimulation (DBS) implantation targeting the ventral intermediate nucleus of the thalamus in June 2021. The patient presented with severe postural and action tremor predominantly affecting the right upper limb, resulting in significant impairment of daily functioning and reduced quality of life. Pharmacological treatment had failed to provide adequate symptom control. Preoperative neuropsychological evaluation revealed no cognitive impairment, psychiatric comorbidities, or behavioral disturbances. Brain magnetic resonance imaging (MRI) demonstrated no structural abnormalities. The first patient had a body mass index (BMI) of 21.3 kg/m^2^.

A DBS lead (Model 3387, Medtronic, Minneapolis, MN, USA) was implanted under local anesthesia. Subsequently, a single-channel implantable pulse generator (IPG; Activa SC 37603, Medtronic) was implanted in the left infraclavicular region under general anesthesia. The IPG was secured to the fascia using two anchoring sutures according to the dual-anchor fixation technique. Initial postoperative programming resulted in satisfactory tremor reduction and significant functional improvement (Table 1).

Two years after implantation, the patient reported progressive abnormal mobility of the IPG associated with positional changes and increasing discomfort at the implantation site. Physical examination demonstrated excessive mobility of the device within the infraclavicular pocket without signs of skin erosion, local infection, or palpable hardware disruption. The IPG could rotate approximately 90 degrees within the pocket but could not complete a full rotation. The patient denied intentional manipulation of the implanted device. Despite these symptoms, DBS efficacy remained preserved, and routine impedance measurements were within normal limits during follow-up visits.

Because classical manifestations of TS, such as loss of stimulation efficacy or abnormal impedance values, were absent, TS was not initially suspected, and no preoperative radiological imaging was performed including plain radiographs. Differential diagnostic considerations included postoperative pocket enlargement, partial fixation failure, and mechanical instability of the IPG system.

**Figure 1:**
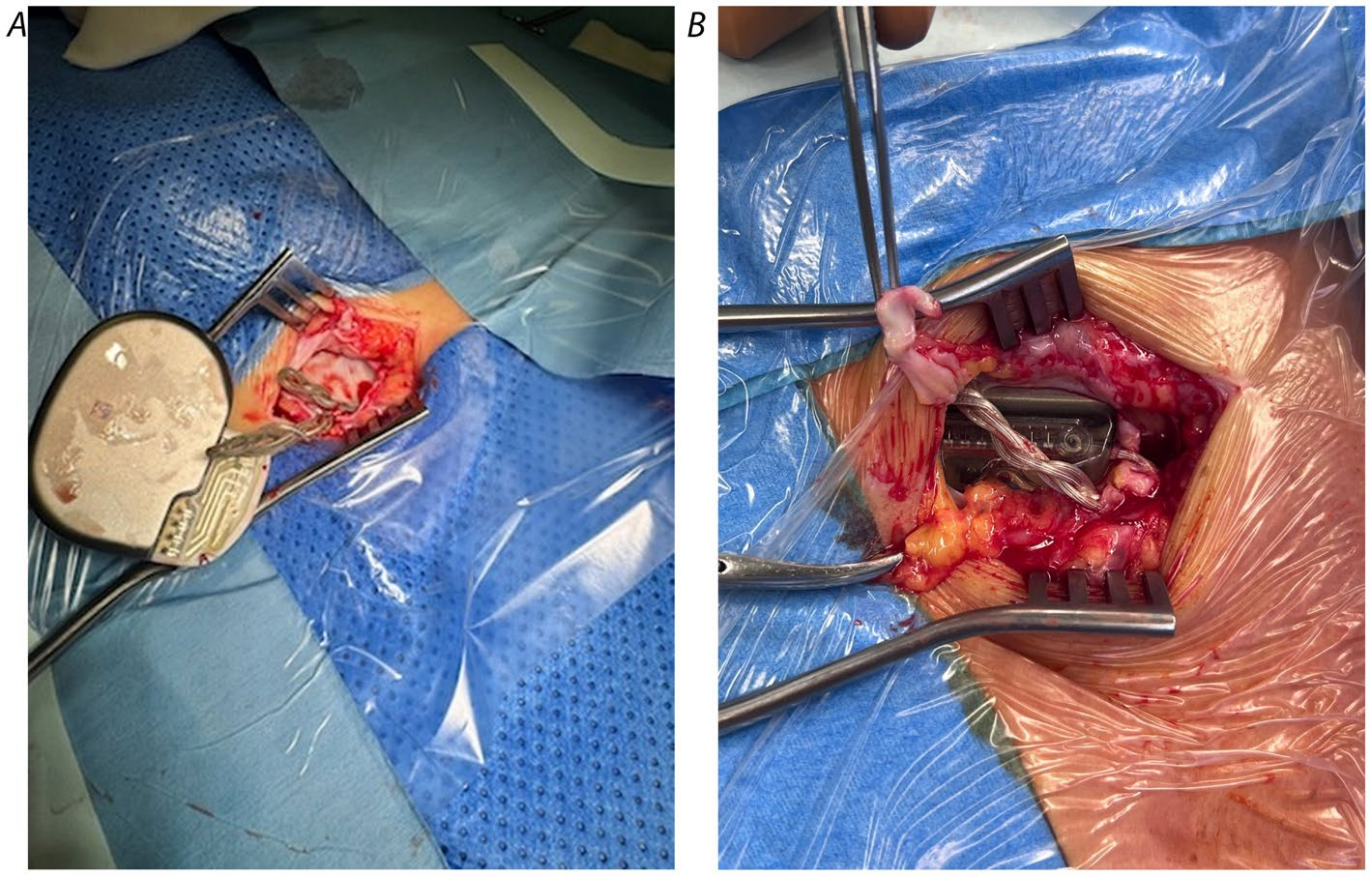
Intraoperative views of severe coiling of the deep brain stimulation (DBS) extension cable observed during implantable pulse generator (IPG) replacement: (A) Case 1; (B) Case 2.

The patient subsequently underwent elective IPG replacement because of battery depletion. During revision surgery, severe twisting and coiling of the extension lead together with disruption of one anchoring suture were identified intraoperatively (Figure 1A). As the extension cable remained structurally intact and impedance values were normal, cable replacement was not required. The extension lead was carefully untwisted, and a new IPG was implanted beneath the pectoralis major muscle within a reduced subpectoral pocket. The device was firmly secured to the fascia using two anchoring sutures. The revision surgery was performed under local anesthesia with slight sedation. There was no indication to replace the untwisted extension lead, as its structural integrity remained intact. Such a procedure would have required a more extensive surgical intervention under general anesthesia, including explanation of the existing extension lead and implantation of a new lead via a newly created subcutaneous tunnel. Given the preserved integrity and proper function of the original extension lead, this approach was deemed unnecessary in the present case.

At 8-month follow-up, the patient reported complete resolution of pain and abnormal IPG mobility. DBS therapy remained clinically effective, and impedance measurements continued to be within normal limits (Table 1).

### 2.2 Case 2

A 69-year-old woman with a 10-year history of medically refractory essential tremor underwent bilateral DBS implantation targeting the posterior subthalamic area. The patient presented with severe postural tremor affecting both upper extremities and disabling head tremor refractory to pharmacological therapy and botulinum toxin injections. Preoperative neuropsychological assessment demonstrated no cognitive impairment or psychiatric disorders. Brain MRI revealed no significant abnormalities. The second patient had a body mass index (BMI) of approximately 22.5 kg/m^2^.

Directional DBS leads (Cartesia™ X, Boston Scientific, Marlborough, MA, USA) were implanted bilaterally under general anesthesia using stereotactic neuronavigation. A dual-channel non-rechargeable IPG (Vercise Genus P16, Boston Scientific) was implanted in the left infraclavicular region and secured to the fascia using two anchoring sutures. Postoperative DBS programming resulted in substantial tremor reduction and improvement in daily functioning (Table 1).

Two months after surgery, the patient noticed abnormal positional mobility of the IPG, particularly while lying on her left side. Physical examination revealed excessive mobility of the device within the subcutaneous pocket without complete rotation or evidence of skin complications. Mild tenderness along the extension cable trajectory was noted; however, no palpable cable fracture was detected. Electrode impedance values remained within normal ranges, and DBS therapy continued to provide stable symptomatic benefit. The patient denied deliberate or unconscious manipulation of the IPG.

Over the following months, the patient developed progressive discomfort and tension along the extension cables despite preserved stimulation efficacy and stable hardware parameters. Six months after implantation, positional changes resulted in approximately 90-degree rotation of the IPG within the pocket, prompting surgical revision. Differential diagnostic considerations included enlargement of the subcutaneous pocket, fixation instability, and early-stage TS.

During revision surgery, severe coiling and twisting of the extension cables were identified intraoperatively (Figure 1B). The twisted extension leads were replaced, and the original IPG was exchanged for a smaller rechargeable device (Vercise Genus R16, Boston Scientific). The new IPG was implanted beneath the pectoralis major muscle in a reduced subpectoral pocket and firmly secured to the fascia using two anchoring sutures. The rationale for replacing the original device with a rechargeable IPG was the relatively large dimensions of the non-rechargeable generator, which were associated with patient-reported discomfort. In addition, the patient requested a smaller and more comfortable device.

At 8-month follow-up, the patient remained free of pain, abnormal device mobility, and hardware-related symptoms. Clinical improvement associated with DBS therapy was maintained, and impedance values remained stable (Table 1).

## 3 Discussion

TS is an uncommon but potentially serious complication of DBS systems, with a reported incidence ranging from 0.07 to 1.3% (Sobstyl et al., 2017). Classically, TS is associated with intentional or unconscious manipulation of the IPG, resulting in hardware rotation, extension lead twisting, lead fracture, abnormal impedance measurements, and eventual loss of stimulation efficacy (Franzini et al., 2018).

Most previously reported cases involved overt hardware dysfunction accompanied by neurological deterioration or sudden recurrence of symptoms (Bayliss et al., 1968; Gelabert-Gonzalez et al., 2010; Israel and Spivak, 2008; Machado et al., 2005). In contrast, both patients presented in this report demonstrated an atypical and clinically silent form of TS characterized by preserved DBS efficacy and normal impedance measurements despite progressive twisting of the extension leads.

The clinical presentation observed in our patients expands the currently recognized spectrum of TS manifestations. Similar asymptomatic cable twisting preceding overt TS has only rarely been described in the literature (Permana et al., 2024).

Importantly, neither patient reported deliberate manipulation of the implanted device, and no psychiatric comorbidities or behavioral disturbances traditionally associated with TS were identified (Franzini et al., 2018). Instead, both patients developed progressive positional IPG mobility and local discomfort despite dual-anchor fixation of the IPG to the fascia. These observations suggest that TS may also develop through passive mechanical mechanisms independent of intentional device manipulation. Repeated postural changes, gravitational forces, chronic micro-movements of the IPG within the pocket, and gradual loosening of fascial fixation may all contribute to progressive rotational stress on the extension leads. In both presented patients, standard non-absorbable sutures were used for IPG fixation, as routinely applied in neuromodulation procedures for essential tremor and related movement disorders. Specifically, polypropylene (Prolene) sutures of 2–0 size were used for anchoring the pulse generators to the surrounding fascial tissue.

Our findings additionally question the absolute protective value of dual-anchor fixation techniques. Previous reports suggested that dual-anchor fixation may significantly reduce the risk of TS and hardware instability (Burdick et al., 2010; Sobstyl et al., 2017). However, in both presented cases, severe extension lead coiling developed despite the use of modern DBS systems and dual-point fascial fixation. In one patient, intraoperative findings demonstrated disruption of one anchoring suture, suggesting that progressive mechanical stress may eventually overcome standard fixation methods. These findings indicate that dual-anchor fixation reduces, but does not completely eliminate, the risk of TS development. Although dual-point anchoring techniques are intended to enhance device stability and reduce the risk of TS, persistent or progressive mechanical stress within the implant pocket may still be transmitted to the fixation sutures, potentially leading to suture loosening or partial failure over time.

An important clinical observation from the present cases is that preserved DBS benefit and normal impedance measurements do not exclude silent TS. In both patients, the diagnosis remained clinically unrecognized until revision surgery because classical indicators of hardware dysfunction were absent. Instead, the predominant manifestations consisted of abnormal IPG mobility, positional discomfort, and tension along the extension cable trajectory. These subtle mechanical symptoms may therefore represent an early “silent” pre-failure stage of TS preceding lead fracture or complete hardware malfunction. Increased awareness of these atypical symptoms may facilitate earlier diagnosis and intervention before irreversible hardware damage or abrupt interruption of neuromodulation therapy occurs.

The therapeutic strategy applied in both patients consisted of revision surgery with subpectoral IPG implantation, reduction of the device pocket, and firm fascial fixation. In one case, untwisting of intact extension lead was sufficient, whereas the second patient required complete extension leads replacement because of severe cable deformation. Both patients achieved durable symptom resolution with stable DBS efficacy during follow-up. Subpectoral IPG placement has previously been advocated as an effective method for reducing excessive device mobility and preventing recurrent TS (Astradsson et al., 2011).

Our observations further support the potential role of subpectoral implantation in patients presenting with abnormal IPG mobility or early signs of hardware instability.

The major strength of this report lies in the presentation of two clinically silent cases of impending TS occurring despite modern preventive fixation techniques and preserved DBS function. The cases highlight previously underrecognized clinical manifestations of TS and emphasize the importance of careful assessment of subtle hardware-related symptoms. In addition, the report provides practical surgical management strategies that resulted in successful long-term outcomes.

Several limitations should also be acknowledged. First, this study describes only two cases, which limits the generalizability of the findings. Second, preoperative radiological imaging was not performed because TS was not initially suspected due to preserved stimulation efficacy and normal impedance values. Consequently, the exact temporal progression of extension lead twisting could not be established. Finally, the biomechanical mechanisms responsible for IPG rotation remain speculative and require further investigation in larger cohorts.

Previous reports highlight various preventive surgical techniques aimed at reducing the risk of recurrent TS and associated DBS lead damage. These include strategies designed to secure the pulse generator and leads more effectively to minimize device rotation and traction (Molle et al., 2022; Liu et al., 2022; Nurimanov et al., 2024).

Early radiological assessment, particularly plain radiography, may play an important role not only in delineating the temporal progression of lead twisting but also in enabling earlier diagnosis and informing clinical decision-making. Subclinical or “silent” manifestations of TS, such as increased generator mobility or mild discomfort at the IPG site, may already represent early indicators of an evolving overt syndrome. In such cases, prompt radiological evaluation should be considered to detect early hardware instability. Although this strategy was not applied in our reported cases, it represents a potentially valuable clinical approach that may help prevent progression to full-blown TS in selected patients.

In conclusion, increased IPG mobility associated with local discomfort may represent an early clinical manifestation of TS even in the presence of preserved DBS function and normal impedance measurements. Clinicians should maintain a high index of suspicion for impending TS in patients reporting abnormal device mobility or positional discomfort despite otherwise normal hardware function. Early surgical revision with subpectoral IPG fixation may prevent progression to overt hardware failure, lead fracture, and interruption of neuromodulation therapy.

## 4 Patient perspective

### 4.1 Case 1

The patient reported that progressive mobility of the implanted device caused increasing discomfort during daily activities and anxiety regarding possible device malfunction. Following revision surgery, she experienced complete symptom resolution and improved physical comfort.

### 4.2 Case 2

The patient described concern related to abnormal movement of the implanted device and tension along the extension cables, particularly during positional changes. After surgical revision, she reported restoration of comfort and confidence in the stability of the DBS system.

## Data Availability

The original contributions presented in the study are included in the article/supplementary material, further inquiries can be directed to the corresponding author.
